# Plant expression systems, a budding way to confront chikungunya and Zika in developing countries?

**DOI:** 10.12688/f1000research.9502.1

**Published:** 2016-08-31

**Authors:** Jaime A. Cardona-Ospina, Juan C. Sepúlveda-Arias, L. Mancilla, Luis G. Gutierrez-López

**Affiliations:** 1 Infection and Immunity Research Group, Faculty of Health Sciences, Universidad Tecnológica de Pereira, Pereira, Colombia; 2 Public Health and Infection Research Group, Faculty of Health Sciences, Universidad Tecnológica de Pereira, Pereira, Pereira, Colombia; 3 Biodiversity and Biotechnology Research Group, Faculty of Enviromental Sciences, Universidad Tecnológica de Pereira, Pereira, Colombia

**Keywords:** Chikungunya, Zika, Molecular farming, Vaccine

## Abstract

Plant expression systems could be used as biofactories of heterologous proteins that have the potential to be used with biopharmaceutical aims and vaccine design. This technology is scalable, safe and cost-effective and it has been previously proposed as an option for vaccine and protein pharmaceutical development in developing countries. Here we present a proposal of how plant expression systems could be used to address Zika and chikungunya outbreaks through development of vaccines and rapid diagnostic kits.

Plant expression systems have been used for the past 26 years for the production of human or animal proteins of biopharmaceutical interest. Antigens, antibodies, and enzymes have been produced, and some of them commercialized using several plant expression platforms ^1^. While certain impediments remain such as community reluctance to accept transgenic products and the strict regulations for approval ^2, 3^, the FDA approval of ELELYSO ^®^ (alfa taliglucerase), a recombinant cerebrosidase for the treatment of Gaucher’s disease, has motivated plant-based biopharmaceutical protein production. These methods are all the more attractive because they are cost-effective, safe and scalable ^4^.

After the arrival of Zika and chikungunya viruses to Latin American countries, they quickly became endemic diseases. They currently pose an acute and chronic burden for health systems and represent a diagnostic challenge in areas where those infections co-circulate with dengue and other febrile-illnesses ^5, 6^. Clinical diagnosis frequently is difficult given the similar clinical features with other viral infections such as dengue. However, the laboratory confirmation of Zika, dengue or chikungunya infection is important because each one has different implications for follow-up both in the short and long term. Diagnosis of acute, symptomatic infection is typically achieved through pathogen detection by virus isolation or qRT-PCR, Serology may be helpful later in the acute illness, but requires convalescent sampling in many cases and comes at a significant cost for healthcare systems. For this reason, confirmation is not recommended for the general population and has been restricted to specific cases. On the other hand, prevention of infection has been in the spotlight for policy makers. There are Zika and chikungunya vaccines under development, but current vaccine production is compromised by reduced capacity of vaccine manufacturers and substantial unmet needs for investment ^7^.

Developing countries have been the most affected worldwide with these vector-borne diseases, and plant-based expression platforms have been proposed as a biotechnological tool to address the vaccine development challenge ^8^. Plants could be used as bio-factories for the production of antigens, for both rapid diagnostic test design and vaccine production. Plant platforms operate at a small fraction of the cost (0.1% to 10%) of other expression systems like bacteria or mammalian cells ^9^. Additionally, it has higher protein yield, lower contamination risk, lower storage cost, ability to assemble complex proteins with minor glycosylation differences, as well as high product quality, safety, and scalability ^9, 10^.

Although post-translational modifications have been a concerning issue, plant-derived vaccines can elicit protective immune responses ^11, 12^. Glycoengineering allows modification of protein glycosylation patterns in order to improve immunogenicity. Additionally, plant derived polysaccharides have been proposed as adjuvants and vehicles, further highlighting plants as a biofactory for antigen production ^10, 11^. Finally, viral antigens produced in plants have been used to target other arboviruses like the West Nile virus ^11^.

In this context, a research agenda to assess the production of pharmaceutical proteins through plant molecular farming seems like a possible scenario to deal with current arboviruses epidemics. At first, candidate proteins should be defined. These proteins should be highly conserved and highly immunogenic. Importantly, antigen similarity between flaviviruses like dengue, yellow fever and Zika viruses has limited target antigen selection, for both vaccine and diagnostic test design for Zika, because of cross-reactivity and the risk of antibody dependent enhancement of infection ^13^. Regarding chikungunya virus, the envelope glycoprotein E2 has been studied for both vaccine and rapid diagnostic test design, and even the use of plant produced virus like particles has been proposed as candidate for vaccine production ^14^ ( Table 1).

**Table 1.** Possible protein targets for expression in plant systems for chikungunya vaccine or rapid diagnostic test developing.

Target protein	Protein characteristics	Rapid diagnostic test	Vaccine development	Advantages	Disadvantages	Reference
E1	Protein type: Surface glycoprotein; Function: Virus entry	X	X	Produce neutralizing antibodies response	Plant glycosilation pattern could modify immune response*	15
E2	Protein type: Surface glycoprotein; Function: Viral attachment	X	X	Produce neutralizing antibodies response	Plant glycosilation pattern could modify immune response*	16, 17
nsP2	Cysteine protease with two separate domains with helicase and protease activity		X	Could be used as adjuvant for glycoproteins based vaccines	Reduced immune response induction. Greater genetic diversity	18
E3-E2-6K-E1	Envelope poly-proteins		X	Produce neutralizing antibodies	Large cloning vector size, complex assembly and purification*	19
C-E3-E2-6K-E1	Virus like particles		X	High immunogenicity. Produce neutralizing antibodies that have proved protection against wild-type virus	Large cloning vector size, complex assembly and purification*	20– 22
Neutralizing Monoclonal Antibodies	IgG monoclonal antibodies against E1, E2 or C		X	Could be used in passive immunization	Difficult production in commercial scale, complex cloning vector assembly	23, 24

*Plant glycosylation could both enhance or limit immune response.

In addition, the plant expression system should be carefully selected. It is important to note that not all plants can be transformed, and phenolic compounds produced by plants and some of its secondary metabolites could make the purification of the desired protein difficult. Furthermore, the risk of contamination of other crops by the spread of transgenic pollen must be monitored according to the transformation method and plant species used for it. Transformation protocols in *Nicotiana benthamiana, N. tabacum,* and *Solanum tuberosum* have been used most commonly ^9^.

Because of its scalability, efficiency and effectiveness, transformation using *A. tumefaciens* has been the preferred method for biopharmaceutical protein production. This method does not require special equipment like the gene gun, it allows a more precise and selective transgene insertion, and results in lower tissue damage and thus higher available biomass for protein production. Using this method both transient and stable transformation is obtained. In recent years, the method of agroinfiltration for transient plant transformation is preferred because of its potential to be systematized and provide an adequate yield of protein in the short-term ^4, 9^.

In conclusion, plant expression systems of heterologous proteins are a feasible strategy for vaccine development and rapid diagnostic kit design. Additionally, it could enable developing countries to address the challenge of current arboviruses epidemics, both in improving diagnostics as well as increasing primary prevention. The development of a molecular plant farming research agenda seems as a worthy solution to empower research in developing countries. It will permit every country to take advantage of its own natural resources in an individualized manner to deal with its own epidemiologic challenges.
