# Supplementary material for: Plant expression systems, a budding way to confront chikungunya and Zika in developing countries?
Source: F1000Res. 2016 Aug 31;5:2121. [Version 1] doi: 10.12688/f1000research.9502.1 (PMC5054818; doi:10.12688/f1000research.9502.1)
Supplement: Peer review history

# Peer review: Referee response for version 1

**DOI:** 10.5256/f1000research.10235.r16839

**Published:** 2016-10-06

**Authors:** Fredrik Pettersson

**Affiliations:** 1 Department of Cell and Molecular Biology, Uppsala University, Uppsala, Sweden

The authors discuss an interesting concept of using plants as vehicles for the production of vaccines and pharmaceutically important proteins. Although it is a promising field with some successful examples already existing, I get the impression from the text that it may lead to a quick solution for the vaccine development targeting the current Zika and chikungunya outbreaks. However, given the time for the development and testing needed before such a vaccine could be administered to a larger population I cannot foresee that this will happen in the near future. Maybe this can be rephrased to reflect this. Furthermore, the skeptical or negative opinion against transgenic crops existing in many countries may also affect the medical substances in question here, especially if targeting edible vaccines. As this may be a larger obstacle than the actual technological challenges, this could have been discussed further.

I have read this submission. I believe that I have an appropriate level of expertise to confirm that it is of an acceptable scientific standard.

# Peer review: Referee response for version 1

**DOI:** 10.5256/f1000research.10235.r16476

**Published:** 2016-09-28

**Authors:** Antonio Carlos Albuquerque Bandeira

**Affiliations:** 1 Faculty of Technology and Science (Faculdade de Tecnologia e Ciências), Salvador, Brazil

Dr Ospina gives us a clear insight on the plant platforms for producing antigens for both diagnosis and for vaccine development in Chikungunya and Zika. It enables a large scale production with lower costs and should be evaluated in countries with resource-limiting conditions.

I have read this submission. I believe that I have an appropriate level of expertise to confirm that it is of an acceptable scientific standard.
